# The role of extracellular Tau in the spreading of neurofibrillary pathology

**DOI:** 10.3389/fncel.2014.00113

**Published:** 2014-04-23

**Authors:** Miguel Medina, Jesús Avila

**Affiliations:** ^1^Centro de Investigación Biomédica en Red de Enfermedades Neurodegenerativas (CIBERNED)Madrid, Spain; ^2^Centro de Biología Molecular “Severo Ochoa” CSIC-UAMMadrid, Spain

**Keywords:** Alzheimer, exosomes, vesicles, neurodegeneration, propagation, spreading, tau, tauopathies

## Abstract

The microtubule-associated protein (MAP) tau plays a critical role in the pathogenesis of Alzheimer’s disease (AD) and several related disorders collectively known as tauopathies. Development of tau pathology is associated with progressive neuronal loss and cognitive decline. In the brains of AD patients, tau pathology spreads following an anatomically defined pattern. Mounting evidence strongly suggests that accumulation of abnormal tau is mediated through spreading of seeds of the protein from cell to cell and point at the involvement of extracellular tau species as the main agent in the interneuronal propagation of neurofibrillary lesions and spreading of tau toxicity throughout different brain regions in these disorders. That would support the concept that pathology initiates in a very small part of the brain many years before becoming symptomatic, spreading progressively to the whole brain within 10–20 years. Understanding the precise molecular mechanism underlying tau propagation is crucial for the development of therapeutics for this devastating disorder. In this work, we will discuss recent research on the role of extracellular tau in the spreading of tau pathology, through synaptic and non-synaptic mechanisms.

## Introduction

A common pathological feature of many neurodegenerative diseases, including Alzheimer’s disease (AD), Parkinson’s disease (PD), Huntington’s disease (HD), amyotrophic lateral sclerosis (ALS) or prion diseases, among others, is the abnormal deposition of proteins in the brain. Among these pathological proteins, the MAP tau forms intraneuronal filaments in a spectrum of neurological disorders collectively known as tauopathies.

Tau protein is a MAP that under physiological conditions regulates microtubules (MT) assembly, dynamic behavior, and spatial organization, and has also been shown to regulate the axonal transport of organelles, including mitochondria. The gene encoding tau protein *MAPT* is located on chromosome 17q21.3, spans approximately 150 kb and consists of 16 exons (Pittman et al., 2006) from which six major isoforms are expressed in adult brain through alternative splicing (reviewed in Andreadis, 2012). The interaction between tau and tubulin is mediated by four imperfect repeat domains (encompassing 31–32 residues) encoded by exons 9–12 (Lee et al., 1989). Alternative splicing of exon 10 results in the production of isoforms containing 3 or 4 binding domains (3R and 4R tau) (Himmler et al., 1989).

Adult human brain contains equal amounts of 3R and 4R isoforms whereas foetal brain, however, only expresses 3R tau, demonstrating developmental regulation of exon 10 splicing (Goedert et al., 1989). Different brain regions also differ in the relative levels of 3R and 4R isoforms with granule cells in the hippocampal formation reported to have only 3R tau. Disturbances, usually increases, in the 3R/4R ratio are a common feature in most neurodegenerative tauopathies. Furthermore, morphological differences exist among different diseases or disease types as different tau isoforms are accumulated in diseased brains, namely, six tau isoforms in AD, 3R tau isoforms in Pick’s disease, and 4R tau isoforms in profressive supranuclear palsy (PSP) and cortical basal degeneration (CBD; Goedert and Spillantini, 2011). Interestingly, a recent study has shown that the 4R/3R ration may have been underestimated in AD brains when compared with PSP or CBD, presumably due to extensive deamidation at Asn279 (Dan et al., 2013).

Within neurons, tau is predominantly found in axons (Hanger et al., 2009) as a highly soluble phosphoprotein (Iqbal et al., 2009). Phosphorylation is also developmentally regulated, with a high tau phosphorylation level during embryogenesis and early development, when only the shortest of the isoforms is being expressed. By contrast, adult brain expresses all six isoforms with relatively reduced phosphorylation levels compared with the foetal one (Liu et al., 2007).

The key discovery directly involving tau protein in neurodegeneration and dementia came from the finding that highly penetrant, dominant mutations in the *MAPT* gene encoding tau cause an inherited form of frontotemporal dementia and parkinsonism (Hutton et al., 1998; Poorkaj et al., 1998; Spillantini et al., 1998). A number of neurodegenerative disorders present prominent tau pathology in the CNS, predominantly within the neuronal compartment, but also within glial cells. Because of this shared histopathological feature, they are referred collectively as tauopathies, although they constitute a group of etiologically heterogeneous, clinically and neuropathologically overlapping disease entities (Ballatore et al., 2007; Spillantini and Goedert, 2013). In tauopathies, the intracellular soluble tau forms filamentous structures of aggregated, hyperphosphorylated tau, which are associated with synaptic loss and neuronal death. The occurrence of fibrillar tau inclusions in tauopathies strongly supports a key role in the observed clinical symptoms and pathology.

Further insights into the overlapping pathogenic and etiologic aspects of the discrete diseases will help to design (perhaps common) disease-modifying treatment strategies (Medina, 2011). To achieve that goal however, it is critical to understand the normal biological roles of tau, the specific molecular events that induce tau to become neurotoxic, the biochemical nature of pathogenic tau, the means by which pathogenic tau exerts neurotoxicity, and how tau pathology propagates.

As mentioned, the recognition of the MAP tau as a key player in the pathobiology of human neurodegenerative diseases has led to major efforts to understand its biological and pathological function(s). This has resulted in an improved understanding of tau cellular functions beyond its classical role in stabilizing MT (Morris et al., 2011) to unveil novel physiological tau functions that may also play a role in pathogenesis. Such functions include axonal transport (Terwel et al., 2002; Rodríguez-Martín et al., 2013), neuronal polarization (Caceres and Kosik, 1990; Dawson et al., 2010), axonogenesis (DiTella et al., 1994; Klein et al., 2002; Belkadi and LoPresti, 2008), interactions with the plasma membrane (Brandt et al., 1995; Lee et al., 1998; Maas et al., 2000), signal transduction (Lee et al., 2004; Ittner et al., 2010) and cell cycle (Andorfer et al., 2005). Furthermore, despite lacking an identified nuclear localization signal, tau has also been reported in nuclei in a number of cell lines (Loomis et al., 1990; Wang et al., 1993) and human brain (Brady et al., 1995) where it may play a role in DNA protection (Sultan et al., 2011).

## Extracellular Tau

It has been over 20 years since the original report that intracellular tau levels are increased in the brains of AD patients when compared to non-demented controls (Barton et al., 1990; Khatoon et al., 1992). This increase in the amount of tau could be toxic to neurons since a reduction in the amount of intracellular tau has indeed a protective effect in mouse models of neurodegeneration (Rapoport et al., 2002; Roberson et al., 2007) and it has been suggested that reducing tau levels may be therapeutically beneficial (Götz et al., 2013). However, we must be cautious since other studies in similar tau-deficient mice point in the opposite direction, suggesting that loss of tau function can actually lead to neurodegeneration (Dawson et al., 2010).

Little is known about how tau synthesis is regulated although some factors such as fibroblast growth factor (Tatebayashi et al., 1999), Dyrk1A (Qian et al., 2013), or the haplotype H1 have been involved in increased synthesis whereas the miRNA-34 family (Dickson et al., 2013) seems to downregulate tau levels.

Conventional wisdom has suggested that the presence of tau in the brain parenchyma or in the cerebrospinal fluid (CSF) is a consequence of tau protein being released from dead cells. However, this has recently been challenged by a number of studies showing extracellular tau being released from cell lines and neurons via multiple pathways, strongly supporting the notion that secretion of tau protein may be an important biological function of tau protein, especially in disease. Despite the fact that tau lacks a signal sequence a number of reports have now shown that tau is released into culture medium from neuroblastoma cells, tau-expressing non-neuronal cells, induced pluripotent stem cell-derived human neurons, and mouse primary neurons (Kim et al., 2010; Shi et al., 2012). Thus, tau has been reported to be secreted unconventionally in naked form (Chai et al., 2012) or associated to exosomes (Saman et al., 2012) and/or other membrane vesicles (Simón et al., 2012a). Since increased tau cellular levels are detrimental, secretion has been proposed as a mechanism to eliminate the excess of tau protein thereby avoiding its toxicity (Simón et al., 2012b). Interestingly, while full length tau has been detected in the extracellular space, C-terminal cleavage of tau has been shown to enhance its secretion (Plouffe et al., 2012) which could have pathological relevance since some truncated tau species appears to be characteristic of particular tauopathies whereas other tau fragments may be common to several tauopathies (Hanger and Wray, 2010; Kovacech et al., 2010).

Extracellular tau has also been detected in the brain interstitial fluid of both wild-type and P301S tau-expressing mice in microdialysis studies (Yamada et al., 2011), as it has also been the case in patients following severe traumatic brain injury (Marklund et al., 2009; Magnoni et al., 2012). Actually, exosomal tau secretion has been suggested to account for the elevated CSF tau levels typically observed in early AD (Saman et al., 2012). Interestingly, tau mutations that are associated with the development of tauopathy appears to reduce tau release (Karch et al., 2012). Interestingly, physiological secretion of endogenous tau by cortical neurons appears to be regulated by neuronal activity, as tau release is enhanced by glutamate receptor stimulation induced by the agonist *S*-AMPA (Pooler et al., 2013). This process is calcium-dependent and modulated by phosphorylation and released tau is present in a relatively dephosphorylated state, compared to that of intracellular tau.

**Figure 1:**
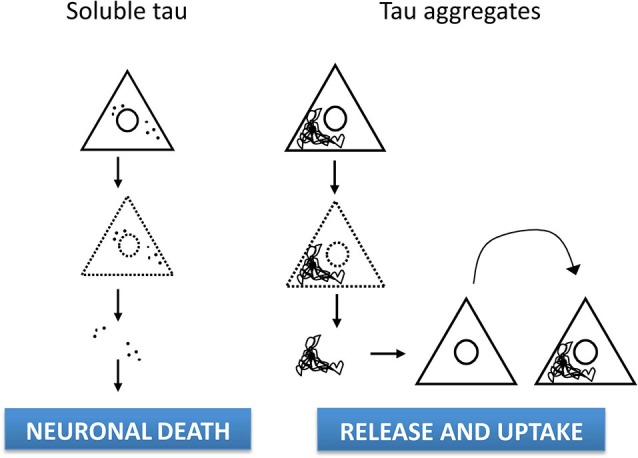
**Cell-to-Sources of extracellular tau and -cell spreading**. The established view has long considered the presence of tau in the brain parenchyma or in the CSF as a consequence of tau protein being released after cell lysis. However, extracellular tau appears to result from a physiological process independent of cell death, as tau being can be released from cell lines and neurons via multiple pathways, either in naked form or vesicle-associated. In addition, tau misfolding in diseased brain leads to abnormal conformations of tau that can be taken up by surrounding neurons. Thus, pathological progression could involve transmission of tau protein through a prion-like mechanism resulting in neurodegeneration in susceptible brain regions.

Thus, increasing evidence point out to extracellular tau as a physiological process independent of cell death (Figure 1), although the precise relationship between tau release under physiological conditions and the propagation of pathology in AD and other tauopathies remains to be determined.

Tau can be toxic when applied extracellularly to cultured cells (Gómez-Ramos et al., 2006; Kopeikina et al., 2012). Several mechanisms for internalization of tau has been proposed, such as internalization of soluble, uncoated (“naked”) tau via receptor-mediated endocytosis (Gómez-Ramos et al., 2009), dynamin-driven endocytosis of non-fibrillar, soluble tau aggregates (Wu et al., 2013) or even actin-dependent, proteoglycan-mediated macropynocytosis (Holmes et al., 2013). Furthermore, it has been suggested that extracellular tau might provoke a receptor-activated increase in intracellular calcium through M1/M3 muscarinic receptor stimulation (Gómez-Ramos et al., 2008; Díaz-Hernández et al., 2010) and that such receptor activation could lead to endocytosis of extracellular tau. Remarkably, tau phosphorylation could inhibit its interaction with M1/M3 receptors and it has been proposed that such alterations might be involved in the transmission of tau pathology (Simón et al., 2013).

The discovery of extracellular tau as a physiological process that is independent of cell death (Pooler et al., 2013), indicates that tau release does not occur only as a result of reduced neuronal viability, and therefore that the increased tau observed in interstitial fluid and CSF in tauopathies may not be due solely to tau release from dying neurons (Yamada et al., 2011; Nedergaard, 2013). It is worth mentioning that tau phosphorylation at threonine 181 and total tau levels in CSF are considered useful biomarkers of neuronal degeneration or injury in the recent National Institute on Aging and Alzheimer Association (NIA-AA) revised criteria for the diagnosis of AD (Jack et al., 2012).

## Propagation of Tau pathology

Development of tau pathology is associated with progressive neuronal loss and cognitive decline. In the brains of AD patients, tau pathology propagates following an anatomically defined pattern described by the neuropathological Braak sequential staging (Braak et al., 2011). As a matter of fact, recent NIA-AA guidelines recommend the assessment of Braak and Braak staging of neurofibrillary degeneration as part of the so called “ABC score” for the neuropathological diagnosis of AD (Montine et al., 2012).

The originally staging system (Braak and Braak, 1991) defined six stages based on the presence and density of characteristic argyrophylic inclusions (neurofibrillary tangles (NFT), neuropil threads) in the medial temporal lobe and several brain isocortical regions. This system was subsequently adapted by the authors for routine use in paraffin-embedded tissue based on tau immunohistochemistry (Braak et al., 2006). Stages I–II (transentorhinal) correlate with the lengthy preclinical phase of the disease; whereas stages III–IV (limbic) do so with mild cognitive impairment (loss of episodic memory) or mild dementia; and advanced V–VI stages (isocortical) usually correspond to cases with moderate to severe dementia. Accurate staging of AD-related tau-positive pathology may be particularly important in the classification of preclinical disease and in the identification of atypical AD phenotypes. The above mentioned NIA-AA guidelines allow for standardization for diagnostic and research purposes.

Clinicopathological studies show that tau pathology progression from the entorhinal cortex through the hippocampus and into the limbic and association cortex is the main neuropathological variable that correlates with the clinical cognitive status of the patient (Arriagada et al., 1992; Nelson et al., 2012). Whether that pattern of accumulation reflects cell-to-cell spreading of disease, or simply successive involvement of differentially resistant neuronal populations, has been a matter of debate in recent years. Recent evidence from human studies suggests that tau pathology is actually linked to existing networks of neuronal connectivity. Thus, rather than diffuse, random, or confluent, tau pathology would target specific large-scale distributed networks that in the healthy brain feature convergent intrinsic functional and structural covariance (Seeley et al., 2009). However, the precise molecular and cellular mechanisms by which tau propagates and neuronal networks degenerate are still unknown.

Increasing evidence suggests that synaptic dysfunction is a key pathophysiological hallmark in neurodegenerative disorders, including AD which has been indeed considered a synaptopathy (Selkoe, 2002; Sheng et al., 2012), as synapse density best correlates with the cognitive decline observed in patients. Long regarded primarily as an axonal protein, when hyperphosphorylated tau also accumulates in the somatodendritic compartment during AD (Ballatore et al., 2007). Actually, tau mislocation in dendritic spines has been proposed to lead to synaptic dysfunction by various mechanisms, including regulating the amount of glutamate receptors in spines (Hoover et al., 2010), interacting with post-synaptic signaling complexes, targeting of synaptic mitochondria (Pooler et al., 2014) or destabilizing dendritic spines and dendritic arbor (Koleske, 2013). Presence of tau in the synapse in healthy brains suggests a role for tau in regulating normal synaptic function whereas during neurodegeneration, tau synaptotoxicity seems to be related to soluble forms rather than insoluble aggregates (Pooler et al., 2014).

**Figure 2:**
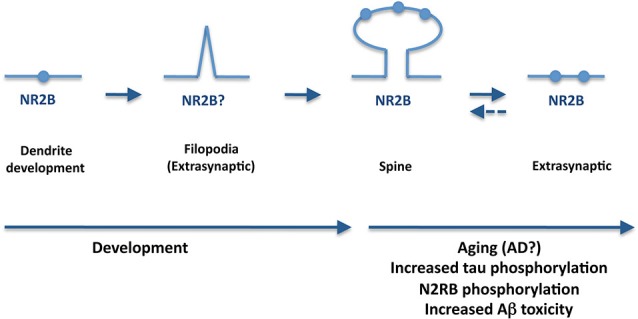
**A potential role for tau in synapsis during aging and AD**. During AD, tau is hyperphosphorylated and mislocates to the axonal compartment. Furthermore, tau appears essential for Aβ-induced synaptotoxicity whereas extrasynaptic NR2B-containing NMDA receptors are required for tau-induced neurodegeneration. An increase of extrasynaptic receptors with aging and AD may also explain the progression of the disease.

On the other hand, emerging evidence strongly suggests that tau is essential for Aβ-induced synaptotoxicity (Ittner et al., 2010), a process that may involve EphB2, and NMDA receptors (Cissé et al., 2011; Sheng et al., 2012). Furthermore, studies in mouse organotypic hippocampal slice cultures from amyloid precurssor protein transgenics have demonstrated that extrasynaptic NR2B-containing NMDA receptors are required for tau-induced neurodegeneration (Tackenberg et al., 2013). Could then tau play a role in the transition between synaptic and extrasynaptic NMDA receptors? Although we do not have a definitive answer to that, the improved NMDA receptor antagonist nitromemantine protects against Aβ-induced synaptic dysfunction (Talantova et al., 2013). Nitromemantine selectively inhibits extrasynaptic over synaptic NMDA receptor activity (Kaufman et al., 2012), thus preventing the toxic effect of the activation of extrasynaptic NMDA receptors. Aβ binding to synaptic or extrasynaptic receptors may lead to different signaling and consequences (protection or death) (Li et al., 2011). Increase of extrasynaptic receptors with aging and AD may also explain the progression of the disease (Figure 2).

Interestingly, recent *in vivo* studies in tauopathy transgenic mouse models expressing human mutant tau specifically in the entorhinal cortex have shown relocation of tau from axons to the somatodendritic compartment as well as propagation of tau pathology to regions outside the entorhinal cortex, strongly suggesting a trans-synaptic mechanism of spreading of pathology through anatomically connected neuronal networks (de Calignon et al., 2012; Liu et al., 2012). These findings have been further supported by more recent neuropathological studies in post-mortem brains from argyrophylic grain disease (AGD), a sporadic tauopathy mainly involving the medial temporal lobe and the limbic region (Ferrer et al., 2008). This pathology exhibits a short number of closely related tau-positive inclusions and a highly homogeneous pattern of distribution and progression of pathology along several regions of the medial temporal lobe with known connectivity between them and with extra-temporal areas of involvement, leading to its proposition as a natural model for studying tau propagation in human brain (Rábano et al., 2014).

Recently, release and subsequent uptake of tau fibrils that directly contact native protein in recipient cells have been shown to mediate propagation of tau misfolding among cells, at least *in vitro* (Frost et al., 2009; Kfoury et al., 2012). Remarkably, intracerebral inoculation of synthetic preformed tau fibrils induced NFT-like inclusions that propagated from injected sites to connected brain regions in a time-dependent manner (Iba et al., 2013). Furthermore, conformation-specific trans-cellular propagation of tau fibrils after release into the extracellular space and subsequent triggering of aggregation in recipient cells by contacting native protein has been show in co-culture experiments (Kfoury et al., 2012). Thus, newly aggregated intracellular tau can transfer between co-cultured cells (Figure 1), thus providing a mechanism for tau-targeted immunotherapies as therapeutic strategy for AD and tauopathies (Gu and Sigurdsson, 2011; Medina, 2011; Golde et al., 2013). Actually, it has been suggested that the most likely mechanism of action for anti-tau antibodies is targeting tau released from cells (Yanamandra et al., 2013). The recent development of imaging-based biomarkers (Maruyama et al., 2013) will enable to track the progression of tau pathology in living patients and greatly facilitate the early phase testing of tau immunotherapy and other tau-based therapeutic strategies.

## Conclusions

In summary, we have highlighted recent developments in tau biology relevant to AD and tauopathies. It has become increasingly clear that, apart from the well-established intracellular functions of tau in microtubule stabilization and axonal transport, intracellular and extracellular tau most likely have important signaling roles that could contribute to the neurodegenerative process in AD and related tauopathies. Furthermore, the presence of tau in synaptic regions of healthy brain suggest that tau may play a role in the regulation of normal synaptic function. In addition, recent studies have suggested that misfolding of tau in diseased brain leads to abnormal conformations of tau that can be taken up by surrounding neurons. Thus, pathological progression could involve transmission of tau protein through a potential prion-like, seeding mechanism resulting in neurodegeneration in susceptible brain regions. However, insufficient evidence exists yet to reliably determine whether there is a direct relationship between the recent identification of a physiological role for extracellular tau and the impairments in tau function associated with disease.

Key questions still remain open, such as the neuronal selectivity, the nature of the extracellular tau species involved, or the precise seeding/templating mechanisms, among many others. More research is needed to identify disease mechanisms driving release and propagation of tau pathology and to determine the impact of extracellular tau on cognitive decline during neurodegeneration. The observation that misfolded tau can be secreted and taken up by adjacent neurons calls for the development of novel strategies to block the propagation of tau pathology in the brain. Despite the fact that the presence of extensive tau pathology is central to the disease, tau-based therapeutic strategies have received little attention until recently (Medina and Avila, 2014). Next few years will certainly bring new insights into the cellular mechanisms underlying tau secretion and uptake, likely identifying novel therapeutic approaches intended to interfere early on in the process of propagation of tau pathology.
